# Management and Outcome of Cardiac and Endovascular Cystic Echinococcosis

**DOI:** 10.1371/journal.pntd.0001437

**Published:** 2012-01-03

**Authors:** Marta Díaz-Menéndez, José Antonio Pérez-Molina, Francesca Florence Norman, Ana Pérez-Ayala, Begoña Monge-Maillo, Pilar Zamarrón Fuertes, Rogelio López-Vélez

**Affiliations:** Tropical Medicine and Clinical Parasitology, Infectious Diseases Department, Ramón y Cajal Hospital, Instituto Ramón y Cajal de Investigación Sanitaria (IRYCIS), Madrid, Spain; Universidad Peruana Cayetano Heredia, Peru

## Abstract

**Background:**

Cystic echinococcosis (CE) can affect the heart and the vena cava but few cases are reported.

**Methods:**

A retrospective case series of 11 patients with cardiac and/or endovascular CE, followed-up over a period of 15 years (1995–2009) is reported.

**Results:**

Main clinical manifestations included thoracic pain or dyspnea, although 2 patients were asymptomatic. Cysts were located mostly in the right atrium and inferior vena cava. Nine patients were previously diagnosed with disseminated CE. Echocardiography was the diagnostic method of choice, although serology, electrocardiogram, chest X-ray, computed tomography/magnetic resonance imaging and histology aided with diagnosis and follow-up. Nine patients underwent cardiac surgery and nine received long-term antiparasitic treatment for a median duration of 25 months (range 4–93 months). One patient died intra-operatively due to cyst rupture and endovascular dissemination. Two patients died 10 and 14 years after diagnosis, due to pulmonary embolism (PE) and cardiac failure, respectively. One patient was lost to follow-up. Patients who had cardiac involvement exclusively did not have complications after surgery and were considered cured. There was only one recurrence requiring a second operation. Patients with vena cava involvement developed PEs and presented multiple complications.

**Conclusions:**

Cardiovascular CE is associated with a high risk of potentially lethal complications. Clinical manifestations and complications vary according to cyst location. Isolated cardiac CE may be cured after surgery, while endovascular extracardiac involvement is associated with severe chronic complications. CE should be included in the differential diagnosis of cardiovascular disease in patients from endemic areas.

## Author Summary

Cardiac and vascular involvement are infrequent in classical cystic echinococcosis (CE), but when they occur they tend to present earlier and are associated with complications that may be life threatening. Cardiovascular CE usually requires complex surgery, so in low-income countries the outcome is frequently fatal. This case series describes the characteristics of cardiovascular CE in patients diagnosed and treated at a Tropical Medicine & Clinical Parasitology Center in Spain. A retrospective case series of 11 patients with cardiac and/or endovascular CE, followed-up over a period of 15 years (1995–2009) is reported. The main clinical manifestations included thoracic pain or dyspnea, although 2 patients were asymptomatic. The clinical picture and complications vary according to cyst location. Isolated cardiac CE may be cured after surgery, while endovascular extracardiac involvement is associated with severe chronic complications. CE should be included in the differential diagnosis of cardiovascular disease in patients from endemic areas. CE is a neglected disease and further studies are necessary in order to make more definite management recommendations for this rare and severe form of the disease. The authors propose a general approach based on cyst location: exclusively cardiac, endovascular or both.

## Introduction

Cystic echinococcosis (CE) is a zoonotic infection caused by the larval stage of the tapeworm *Echinococcus granulosus*. This parasitic disease occurs in humans (who act as intermediate hosts) when the ova of *E. granulosus* from canine (definitive host) faeces are accidentally swallowed. After ingestion, the parasite crosses the duodenal wall and spreads via the portal and systemic circulation to the liver, lungs and other organs. CE is endemic in Europe, including the Mediterranean area and the Balcanic nations, North and East Africa, India, China, Indonesia and the Southern Cone of the Americas [1].

Hydatid cysts may be found at almost any site of the body, but the liver (60–70%) and lungs (10–15%) are most frequently affected [2]–[4]. The heart is reported to be involved in less than 2% of cases [5]–[9]. Endovascular extracardiac CE is very rare, only a handful of cases have been reported and these are thought to be secondary to the rupture of the primary germinative membrane of cysts located at other sites, mostly in the heart or vena cava (including the intrahepatic portion of the cava), with consequent embolization to the pulmonary or systemic circulation [10]–[16].

We describe 11 patients who were diagnosed and treated for cardiac and endovascular CE. Cases from the literature were also reviewed and recommendations for the management of patients are discussed.

## Methods

### Ethics statement

This was a retrospective analysis, the data were analyzed anonymously and written informed consent was not obtained for individual participants. The database from which patients' information was obtained has been approved by the Ramón y Cajal Hospital's Ethics Committee (Comité Ético de Investigación Clínica, CEIC, Hospital Ramón y Cajal) and is used in accordance with the current laws in Spain (Ley Orgánica de Protección de Datos de Carácter Personal 15/1999) which guarantee patient confidentiality.

A retrospective study was performed involving patients with cardiac and endovascular extracardiac CE, diagnosed and treated over a period of 15 years (January 1995 through December 2009) at the Tropical Medicine & Clinical Parasitology Center of the Ramón y Cajal Hospital in Madrid, Spain.

CE and its location was diagnosed according to echocardiography, computed tomography scan (CT)/magnetic resonance imaging (MRI), electrocardiography, chest X-ray, histological criteria and serology (IHA and ELISA techniques) [17]. Data regarding gender, age, area of origin, clinical presentation, diagnostic methods, surgical and medical management and outcome were analyzed using descriptive statistics.

Also, a systematic search of MEDLINE and EMBASE was carried out (for all published articles until June 2011) using the following terms: cardiac echinococcosis, cardiac cystic echinococcosis, cardiac hydatidosis, cardiac hydatid cyst, cardiac hydatid disease, pulmonary cystic embolism, pulmonary hydatid embolism, caval hydatidosis, cava vein hydatidosis, cava vein echinococcosis, endovascular hydatid disease, endovascular hydatidosis, endovascular echinococcosis. No language, age or gender restrictions were used. Individual articles were included.

## Results

Eleven patients with cardiac and/or endovascular CE were evaluated. All of them were Spanish except one, who was an immigrant Bulgarian child (patient 5). Seven patients lived in rural areas, while the rest came from urban areas, although they had lived intermittently in Spanish rural towns. Demographic characteristics, clinical presentation, CE location (both cardiovascular and extra-cardiovascular), medical and surgical treatment and outcome are summarized in Table 1.

**Table 1:** This shows a description of 11 Patients with Endovascular and Cardiac Cystic Echinococcosis.

Case	Sex/age	Clinical presentation	Cardiovasc. CE location	Inferior vena cava involvement	Extracardiovascular CE location	Surgical Treatment	Chemotherapy*	Outcome/Follow-up**.
1	M/55	Chest pain	IVS	None	Kidney, liver, spleen.	Yes	ALB+PZQ 9 m	Stable/3 y
2	F/66	Dyspnea	LA and pericardium	None	Kidney, spleen, disseminated intraabdominal	Yes	ALB+PZQ 93 m.	Stable (PAIR in liver)/4 y
3	F/65	Asymptomatic	RA	None	NO	No	No	Lost to follow-up/1 y
4	F/44	Chest pain, haemoptysis	RV	None	Lung.	Yes	ALB 54 m.	Stable/14 y
5	M/9	Chest pain	A–V line and pericardium	None	NO	Yes	ALB+PZQ 6 m.	Stable/6 y
6	M/60	Chest pain, dspnea	IVS	None	Lung, liver.	Yes	ALB 9 m.	Death (Cardiac failure after 14 years)/12 y
7	M/19	Chest pain, haemoptysis	RA	Yes	Lung, liver.	Yes	ALB+PZQ 75 m.	Cystic PE. Multiple embolizations./6 y
8	M/64	Chest pain, dyspnea	RA	Yes	Lung, liver, disseminated intraabdominal	Yes	ALB+PZQ 39 m.	Cystic PE. Respiratory insufficiency/11 y.
9	M/32	Chest pain	RA	Yes	Lung, liver	Yes	No	Death (massive systemic vascular dissemination)/1 y
10	M/68	Asymptomatic	RA,	Yes	Lung, liver, disseminated intraabdominal	No	ALB 23 m.	Death (PE after 10 years)/6 y
11	F/60.	Dyspnea	RA,	Yes	Lung, liver, disseminated intraabdominal	Yes	ALB+PZQ 25 m.	Cystic PE. Cystic cholangitis/6 y

M = male. F = Female. IVS = Interventricular Septum. LA = left atrium. RA = Right Atrium. RV = Right ventricle. A–V line: atrium-ventricular line. IVC = Inferior vena cava. ALB = Albendazole. PZQ = Praziquantel. PE = pulmonary embolism. Age in years.

*Time in months.

**Time in years.

The mean age was 49.4 years (sd±20.4; range = 9–68 years, median = 60 years) and four patients were female. Patients' main initial symptoms and signs indicative of cardiovascular disease were chest pain in 7 cases, dyspnea in 4 cases, and haemoptysis in 2 cases. Two patients had no symptoms (diagnosis of cardiovascular CE was made following investigations for other causes). Regarding the cardiovascular examination, only one case (patient 6) had an aortic murmur (grade I/IV). Furthermore, one case (patient 2) had three strokes, epilepsy and intermittent claudication of the lower extremities (a brain CT showed no cerebral CE and carotid ultrasound demonstrated advanced atherosclerosis).

Two cases (patients 3 and 5) showed primary and exclusive cardiac involvement. In the remaining 9 cases (82%) there was concomitant extravascular disease: lung involvement in 1 case (patient 4), lung and liver involvement in 3 cases (patients 7 and 9), and disseminated CE (intraabdominal, kidney, spleen, liver and lungs) in 5 cases (patients 1, 2, 8, 10, 11).

Eosinophilia (>400cells/mm^3^) was present in 6 patients (55%). Serological tests (haemagglutination inhibition and ELISA techniques) were positive for all patients. Electrocardiogram was abnormal in 3 cases: signs of atrial hypertrophy (patient 2), left bundle branch block (patient #1) and right bundle branch block (patient 6) were found. The latter two patients had an interventricular septum cyst. An abnormal cardiac outline was detected on chest X- ray in 2 cases (patients 2 & 5, both patients had a pericardial cyst).

**Figure 1:**
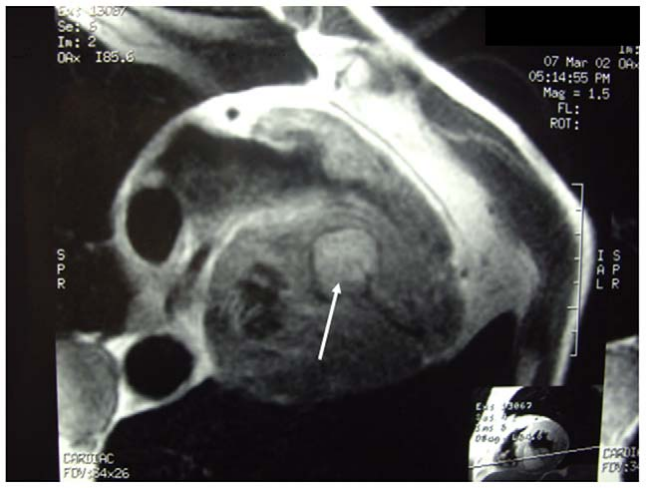
Computed tomography showing CE in intramyocardial interventricular septum (arrow) (patient 1).

Two-dimensional echocardiography was diagnostic in all cases. Cysts in a single location were found in 4 patients: 2 in the interventricular septum (Figure 1), 1 in the right atrium and another in the right ventricle. Cysts in ≥2 cardiovascular locations were present in 7 patients (64%): 5 in the right atrium and vena cava, for 1 patient in the left atrium and pericardium and for another in the atrioventricular area and pericardium. CT and MRI confirmed those findings and also detected a pulmonary hydatid embolism in 5 cases (45%) (patients 7, 8, 9, 10, 11). Data regarding pulmonary artery pressures were not available in most cases.

Histology confirmed the diagnosis of CE in all of the cases where surgical samples were sent for pathological assessment (9/9), according to literature [17].

Management of CE was based on WHO guidelines [18], even though there are no clear recommendations when the disease affects the cardiovascular system. Our approach was as follows:

Nine patients underwent cardiovascular surgery to excise the cysts. Patients 3 and 10 refused surgery, as both were asymptomatic and aged ≥65 years. Cardiopulmonary by-pass was instituted with bicaval cannulation. Standard myocardial protection with cold cardioplegic cardiac arrest was achieved in all cases. Surgical techniques used for cyst removal were radical cystectomy (patients 1, 2, 4, 7, 8, 9, 11), and aspiration associated with capitonnage (Patients 5, 6). In 6 cases, a larvicidal agent (hypertonic saline and/or hydrogen peroxide) was instilled in the area occupied by the cyst. A single intervention was sufficient except in the case of the Bulgarian child (patient 5) with pericardial CE who needed a second intervention (it is possible only partial cystectomy was performed during the first surgery in Bulgaria).

Postoperative complications were documented in 2/9 patients: patient 2 developed atrial fibrillation responsive to antiarrhythmic drugs, and patient 9, who had extensive inferior vena cava involvement and massive hydatid pulmonary embolism, died during surgery secondary to intraoperative cyst rupture. Both the massive antigenic stimuli and pulmonary emboli could be responsible for the death of the patient.

Long-term antihelminthic therapy was prescribed in 9 patients (81.8%). Patient 3 refused medical therapy as she was asymptomatic and patient 9 died during surgery before starting treatment. Drugs used were albendazole 15 mg/Kg/d and praziquantel 40 mg/Kg/d: all patients received albendazole and in 6 cases (67%) this was combined with praziquantel. The mean duration of treatment was 37 months, (Interquartile range 9–64.5 months; median 25 months, range 4–93 months). Three patients developed side effects: 2 (patient 1, 5) had mild transaminase elevation and 1 (patient 11) had mild pancytopenia. All of these effects were reversible after the temporal discontinuation of the drugs.

**Figure 2:**
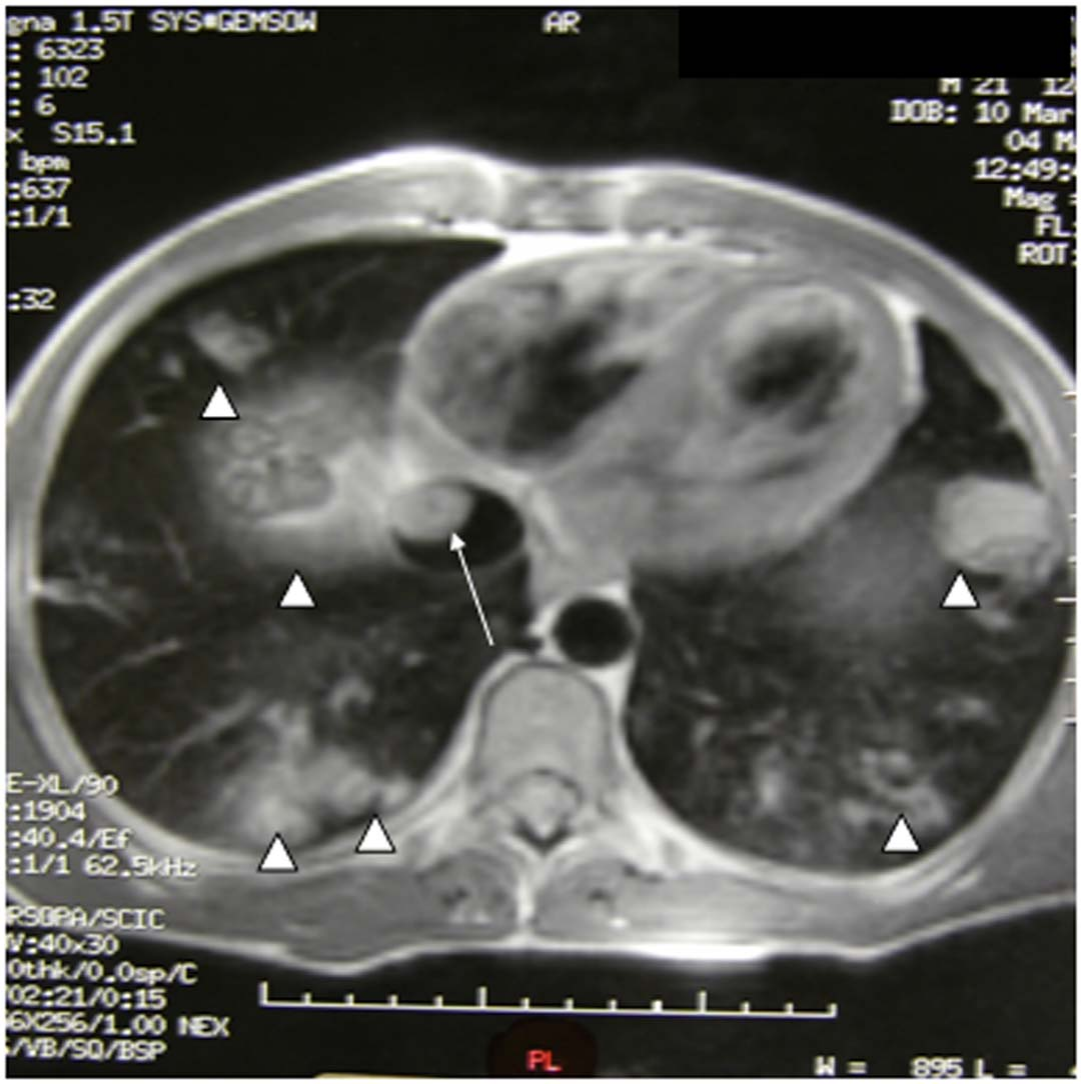
Computed tomography showing IVC cyst (arrow) and multiple CE pulmonary embolisms (arrowheads) (patient 7).

The patients were followed-up for a median period of 6 years (range = 1–14 years). Different outcomes were observed depending on cyst location. Of the two patients with cardiac involvement alone, one (patient 5) remained asymptomatic and stable and the other (patient 3) was lost after 1 year of follow-up. Of the 4 patients with cardiac and disseminated CE (patients 1, 2, 4 and 6), 1 patient was lost to follow up after 3 years (patient 1); for patient 4 cardiac surgery was curative, and the calcified pulmonary cyst remained the same size after 14 years of follow up (CT scan performed annually), with no associated complications. Patient 2 underwent hepatic PAIR (puncture, aspiration, injection, re-aspiration) and remained stable, and patient 6 was stable for the 14 years following diagnosis, but later died due to cardiac failure not related to CE. All 5 patients with cardiac, inferior vena cava and disseminated CE (patients 7–11) developed a pulmonary embolism as a complication (Figure 2). For 4 of the cases CE was the most likely cause of the embolisms, but patient 10, who died 10 years after diagnosis due to embolic pulmonary disease, had multiple risk factors for emboli (bed rest, smoking and chronic venous insufficiency) so the origin/cause of the embolus could not be ascertained. The patient had received albendazole for 23 months with unproven efficacy. Patient 9 died during surgery as mentioned previously. Patient 7 needed multiple embolizations and could have been considered a candidate for transplantation, but previous thoracic surgery would have contraindicated transplantation. Patients 8 and 11, who were heavy smokers, had chronic respiratory insufficiency, diagnosed based on spirometry results. Patient 11 also required cholecystectomy due to CE-associated cholangitis.

Although after reviewing the literature we found more than 200 published cases of cardiac/endovascular CE these are not usually grouped in large series, and are mainly single cases. Table 2 summarizes the case series with 10 or more patients with cardiac CE which have been published in the last ten years [19]–[31]. Table 3 summarizes all reported cases with CE endovascular IVC involvement [32]–[39].

**Table 2:** Cardiac Cystic Echinococcosis series with n≥10 reported in the last 10 years in the literature.

Reference	Period of study country	N° patients	Mean age (years)/Gender (% female)	Cardiac involvement (n)	Concomitanta Extracardiac involvement (%)	Treatment and outcome
Dali, 2000 [19]	1988–1998 Tunisia	17	33 y/47%	Myocardium (16) Pericardium (5)	70.5	17 surgery; 3 early death
Thamaeur, 2000 [20]	1970–1997 Tunisia	45	ND	ND	33.3	45 surgery; 2 early death, 2 recurrence
Khaldoun, 2003 [21]	1983–2001 Tunisia	14	27.7 y/50%	Myocardium (11) Pericardium (2) Multiple (1)	57.1	ND
Akar, 2003 [22]	1984–2001 Turkey	12	31 y/33.3%	Myocardium (12)	ND	12 surgery; 3 early death
Jerbi, 2004 [23]	1991–2003 Tunisia	19	30 y/52.6%	ND	ND	19 surgery; 1 early death
Bouraoui, 2005 [24]	1985–2001 Tunisia	12	40 y/83.3%	Myocardium (9) Pericardium (3)	ND	ND
Elhattaoui, 2006 [25]	1999–2005 Morocco	10	24.7 y/40%	Myocardium (10) Pericardium (4)	70	7 surgery, 2 albendazole; 2 early death.
Orhan, 2007 [26]	1967–2006 Turkey	25	31 y/68%	Myocardium (10) Pericardium (13) Multiple (2)	28	25 surgery; 1 early death, 1 recurrence
Murat, 2007 [27]	1978–2002 China	15	23 y/40%	Myocardium (10) Pericardium (5)	0	15 surgery and albendazole; 1 early death, 4 recurrence
Kabbani, 2007 [28]	1989–2005 Syria	19	25.6 y/57.9%	Myocardium (17) Pericardium (2)	63.2	19 surgery and mebendazole; ND
Tasdemir, 2009 29]	1982–2007 Turkey	10	39 y/20%	Myocardium (11) Pericardium (1) Aorta (1)	10	10 surgery and albendazole; 2 early recurrence
Molavipour, 2010 [30]	1992–2004 Iran	11	25.6 y/63.6%	Myocardium (10) Multiple (1)	ND	11 surgery and albendazole or mebendazole; 1 early death
Tuncer, 2010 [31]	1991–2009 Turkey	13	36 y/46.2%	Myocardium (13)	61.5	13 surgery and albendazole; 1 recurrence

**Table 3:** Inferior vena cava Cystic Echinococcosis cases reported in the literature*.

Reference and country	Age (years)/Gender	CE location	Surgical/medical treatment	Outcome
Aikat, 1978 [32]; India	29 y/M	IVC, RA, Liver and lung	Cystectomy/No medical treatment	Died 6 days after surgery (liver and renal failure)
Landra,1984 [33]; India	NR	IVC, Liver	Cystectomy and caval graft/ND	No recurrence (24 months follow-up)
Ambrosi, 1991 [34]; France	61 y/M	IVC, RA, Liver and lung	Cystectomy/ND	ND
Caballero,1999 [35]; Spain	63 y/F	IVC, RA, Liver	Cystectomy/ALB pre surgery	Died in early postoperative period due to IDC and multiorgan failure
Karunajeewa, 2001 [36]; Australia	65 y/M	IVC, RA, Liver and lung	Cystectomy/ALB 6 w pre and post surgery for 22 w	No recurrence (4 months follow-up)
Sirmali, 2006 [37]; Turkey	33 y/F	IVC, Liver and lung.	Cystectomy and omentoplasty, cyst removal from pulmonary artery (second operation)/ALB 12 w after surgery	No recurrence (16 months follow up)
Meekel,2007 [38]; USA	38 y/M	IVC, Liver	Cystectomy and caval graft/ND	No recurrence (24 months follow up)
Agarwal,2009 [39]; India	13 y/F	IVC, Liver	Cystectomy and capitonnage/ALB 2 w before surgery	No recurrence (6 months follow up)

Note: ALB: Albendazole; CE: cystic echinococcosis; CT scan: computed tomography scan F: female; IVC: inferior vena cava; IDC: intravascular disseminated coagulation; M: male; MRI: Magnetic Resonance Imaging ND: not defined; RA: right atrium; w: weeks.

*Only intraluminal invasion. Cases with vena cava compression and vena cava wall involvement are excluded.

## Discussion

Cardiac and endovascular CE are rare and heterogeneous diseases and therefore management is not standardized. Information from the current series, together with that from other previously published series, may assist in the approach to this complex disease.

Given the slow growth of cysts and assuming exposure during childhood/adolescence, classical CE tends to give symptoms and signs between the third and fifth decade of life [22]. Due to the specific location of cardiac cysts in areas which may result in functional impairment, such as the cardiac conduction system, and because of the limited capacity for expansion (in contrast to organs such as liver or lung), age of onset is younger than for classical CE, with onset of symptoms between the 2nd and 4th decade. This early onset is also seen at other locations, like the eye or the CNS, where even small cysts can rapidly become symptomatic. Cardiac CE rates documented in children under 20 years are around 20% [9], [31] similar to those found in our series: 2/11 cases (18%) were <20 years of age (9 and 19 years, patients 5, 7).

Clinical manifestations depend on the location, number and size of cysts [40]. Most frequently CE presents with thoracic pain or dyspnea, as observed in our series, but the clinical presentation of cardiac CE may range from asymptomatic to life-threatening events. Asymptomatic subjects represent 3–5% of cases and in these cases cysts are not usually located in a critical anatomical site [41]. This may be compared with hepatic CE where up to 50% of cases may be asymptomatic. In these cases, CE may be an incidental finding [42]. It is possible that the number of asymptomatic cases may be underestimated, as more cases with identifiable symptoms may be diagnosed/reported, leading to publication bias. In our series, 2/11 patients (18%) were asymptomatic at the time of diagnosis (patients 3 and 10). Cardiac CE may present with potentially life-threatening events such as cardiac tamponade, heart failure, syncope, arrhythmias, valvular stenosis or regurgitation, pulmonary hypertension, or peripheral embolism [34], [43]. Severe chronic pulmonary hypertension in the setting of recurrent emboli from intrahepatic disease has been reported, as well as acute embolic events resulting in death [42]. Unlike “classic” CE, in which reported mortality rates range between 0.5–4%, in cardiac CE, mortality is 4–10%, usually occurring in the postoperative period [22], [44]. In this series the recorded mortality rate was 27%, but this occurred shortly after diagnosis in only 1 case (patient 9), while for the other two patients (patients 6 and10) death occurred more than 10 years after diagnosis of cardiac involvementand was associated in both with disseminated CE. Cardiac CE usually occurs in the left ventricle or in the interventricular septum. We found a higher prevalence of disease in the right atrium (55%), whereas in the literature reported rates for this location are of 4–7% [9], [40]. This high incidence is probably due to the high incidence of coexisting vena cava involvement that was found in our series.

As with clinical manifestations, complications of cardiac CE also vary according to location of cysts: those on the right side of the heart may fragment and embolize to the lung, causing massive haemoptysis (patient 7) or pulmonary hypertension. Those located on the left side, can produce peripheral emboli [13], [14], [45], [46]: patient 2 was diagnosed with intermittent claudication and stroke, initially attributed to atherosclerosis. Although this patient had documented cardiovascular risk factors, CE could not be ruled out as a cause, as the left atrium was also involved and disease at this site could have been the source of embolisms. The occurrence of pulmonary embolism in cardiac CE is around 6–8% [47]. We have recorded a higher incidence (45%), probably due to the high rates of right atrium and inferior vena cava involvement found in our series.

Cardiac CE is commonly associated with CE disease in other organs [9]. In our series, 45.5% of patients had disseminated disease. Clinical examination is usually unremarkable unless cardiac CE alters valvular function (producing valvular failure or stenosis) [9], [45]. Patient 6 had a heart murmur on auscultation. Even though valvular involvement was not demonstrated by echocardiography, the intraseptal cyst (4,2 cm) protruding into the left ventricle could explain the presence of the murmur.

Cardiac rhythm disturbances are due to involvement of the cardiac conduction system [44], [48]. Three of our patients had ECG abnormalities: two of them had bundle branch block, due to cardiac intraseptal involvement.

Even though serological tests may not be useful in cases of extrahepatic involvement [3] all our patients had positive serological tests (ELISA or HAI). Serology is usually strongly positive in CE cases when there is systemic involvement [49].

An assessment of the heart and main vessels by echocardiography may be recommended for all individuals diagnosed with CE, even if they are asymptomatic. This technique has proven to be highly sensitive in detecting cysts in the heart and surrounding vessels [50], [51].

Both CT and MRI are useful additional imaging techniques to confirm the diagnosis and detect complications (especially in the case of hydatid pulmonary embolisms) [52].

A standardized therapeutic approach may not be possible due to the heterogeneity of cardiovascular CE, and may require the combination of medical and surgical treatment. Benzimidazoles (albendazole, in countries that can afford it, or mebendazole) with or without praziquentel are the basis of pharmacological therapy [53], [17], although the dose and duration of treatment have not been clearly established [54].

Based on this series and published data, the following therapeutic approach may be recommended:

1. If there is cardiac involvement exclusively, surgery may be curative [6]. The technique requires cardiopulmonary bypass and clamping of surrounding vessels to prevent spillage. Instillation of scolicidal substances could be beneficial [8], [30]. In these cases, mortality is mainly related to complications arising from the surgical procedure such as ventricular rupture, fatal arrhythmias,embolisms or massive cyst rupture with anaphylaxis (as in patient 9) [22]. The mortality rate may be higher in centres lacking in expertise or within adequate infrastructure to deal with complex cases. The treatment of cardiac CE in low-income countries is therefore very limited and the prognosis is poor. Surgery is often combined with a short course of pre-intervention prophylaxis [55] with benzimidazoles in order to prevent the spread of the cyst but currently definitive data from clinical trials are lacking [56]. Its use before surgery may even be contraindicated because the pericyst membrane may become more friable and may rupture during surgical manipulation.
2. If there is cardiac involvement and disseminated CE: cardiac surgery may also be required, even though the prognosis is worse due to possible complications at different locations (cyst rupture, fistulae, cholangitis, etc.). Thus, the need for percutaneous treatment (PAIR or PEvac), surgery or other techniques (embolization, etc.) may be conditioned by the possibility of such complications. In this circumstance, the use of albendazole, with or without praziquantel, has shown to be beneficial. Given the poor correlation between serum concentrations of albendazole and parasite viability, the effectiveness of benzimidazoles appears to be more dependant on the duration of treatment than drug blood levels [57]. Therefore, these patients will require long-term treatment to prevent relapses and to slow the growth of existing cysts. For patients 7 and 8 endovascular cysts grew after stopping long-term antiparasitic treatment (treatment discontinued for a mean of 12 months), Treatment was then reinitiated and maintained indefinitely and they remained stable. In our series no relevant side effects were observed despite prolonged treatment, which is consistent with results published in the literature [58], [59].
3. If there is associated vena cava involvement due to CE: it is difficult to draw conclusions regarding management as few cases are documented in the literature. For these patients early and radical management of endovascular disease is proposed in order to avoid associated complications (mainly hydatid embolism). As far as possible, the cyst should be resected and the affected vessel should be reconstructed [60]. Since the majority of pulmonary emboli come from liver cyst rupture near the hepatic veins or inferior vena cava [61], the surgeon should take particular care with vascular structures during surgery of the cysts to prevent spread. If there is a potential risk of vena cava invasion (hepatic cysts close to the vascular bed or in the right atrium) close monitoring of the patient may be of choice. In case of intra-hepatic vascular involvement where there is risk of intra-hepatic inferior vena cava rupture intra-operative caval-caval bypass should be recommended or considered to avoid massive embolization during surgery. In addition, patients should have a pre-operative echo and evaluation of right sided pressures as they may require invasive cardiac monitoring during surgery [62]. Thus, the appearance of lesions may be detected at an early stage, with early removal and clamping of the vessel to prevent the spread of the disease. If the risk of vena cava involvement is very high, surgery may be performed directly on the lesion, although the indication for surgery has not clearly been established in these cases. Whateveroption is chosen, antihelminthic drugs should be used to prevent recurrence.
4. If there is evidence of pulmonary embolism due to CE, surgery will be complex at this location. In cases in which the embolus is already established, early surgery at the source of the emboli may be recommended, combined with indefinite medical treatment.

Although our case series is not large enough to give recommendations regarding follow-up, we suggest indefinite antihelminthic treatment with albendazole in disseminated CE and pulmonary embolism due to CE, and repeated CT scan (preferably CT angiography) in any case surgery cannnot be curative to evaluate changes in the size of the cysts in the heart, vessels and lungs.

Cardiovascular involvement is a rare form of presentation of CE. It presents earlier than classical CE and is associated with complications that may be life threatening. Cardiovascular CE usually requires complex surgery, so that in low-income countries, the prognosis is frequently fatal. CE is a neglected disease and further research is necessary in order to make more definite management recommendations. More prospective studies with a larger number of cases will be needed to define an ideal therapeutic approach and experimental research is mandatory to obtain more effective drugs for treating this devastating disease.

## Supporting Information

Checklist S1 (DOC)
